# Is It T3 Thyrotoxicosis? A Case of Falsely Elevated Tri-Iodothyronine (T3) Levels Leading to a Diagnosis of Multiple Myeloma

**DOI:** 10.1155/2019/5028534

**Published:** 2019-12-25

**Authors:** Nanik Ram, Saira Furqan, Sibtain Ahmed

**Affiliations:** ^1^Department of Medicine, Aga Khan University, Stadium Road, P.O. Box 3500, Karachi 74800, Pakistan; ^2^Department of Pathology and Laboratory Medicine, Aga Khan University, Stadium Road, P.O. Box 3500, Karachi 74800, Pakistan

## Abstract

We are presenting a case of falsely elevated T3 levels in a patient due to interference from monoclonal immunoglobulins. A 56-year-old, clinically euthyroid man referred to the endocrinology clinic of the Aga Khan university, Karachi Pakistan, for possible T3 thyrotoxicosis after thyroid function tests revealed total T3 >12.32 nmol/L (reference range 0.6–2.79), normal TSH, and total T4 level. There was a mismatch in clinical and laboratory parameters and preliminary laboratory results were suggestive of thyroid binding globulin abnormalities. Further evaluation in this context unmasked multiple myeloma. The presence of monoclonal immunoglobulins can lead to assay interference and spurious results. To the best of our knowledge, this is the second case defining the cause of falsely elevated T3 levels, due to assay interferences with binding of T3 only to monoclonal immunoglobulins.

## 1. Introduction

As stated in the text book of clinical chemistry, “the clinical chemist confronted with an immunoassay result which does not appear to fit the clinical picture and perhaps also confronted with an alarmed physician should consider the possibility of a phantom in the immunoassay” [1]. The above statement refers to the spurious results which may be caused by interferences.

For the routine evaluation of thyroid function, analysis of thyrotropin (TSH), thyroxine (T4), and triiodothyronine (T3) are widely used diagnostic methods. However, they are subjected to nonspecific bindings that can interfere with the measurement of these hormones. Literature review revealed only one case till date that descripted the presence of a monoclonal immunoglobulin in the serum or urine, which may exhibit binding to T3 only and lead to falsely elevated results [2].

## 2. Case Presentation

Our patient is a 56-year-old man, who was referred from primary care physician for elevated T3 and possible T3 thyrotoxicosis. For the past few weeks, he had been complaining of fatigue, lethargy, and weight loss. On further evaluation, he had no diarrhea, heat intolerance, skin or hair changes, tremors, visual changes, and palpitations. Furthermore, there was no known personal or family history of thyroid disease. Physical exam was unremarkable except for palpable nodular goiter, and the patient was clinically euthyroid.

In context of fatigue, his primary care physician ordered thyroid function tests, which showed TSH 1.67 *μ*IU/mL (reference range 0.5–8.9), total T3 > 12.32 nmol/L (reference range 0.6–2.79), total T4 4.5 *μ*g/dL (4.6–10.5), free T4 1.08 ng/dL (reference range 0.89–1.76), and free T3 of 2.4 ng/mL (reference range 2.1–4.4). The patient was referred to the endocrinologist clinic for further evaluation The thyroid function test performed on the Siemens Advia centaur analyzer were rechecked, but similar results were obtained. The Siemens Advia centaur T3 assay is a two-site Sandwich/competitive immunoassay using direct chemiluminescent technology. T3 in the patient sample competes with a T3 analog, which is covalently coupled to paramagnetic particles in the solid phase for a limited amount of acridinium ester-labeled monoclonal mouse anti-T3 antibody in the reagent. Furthermore, the same sample was assayed using Abbot Architect Total T3 assay, and results were >8.0 nmol/L. The Architect total T3 assay is a two-step immunoassay to determine the presence of total T3 in human serum and plasma using chemiluminescent microparticle immunoassay technology. This raised the suspicion of a possible endogenous interferent in the sample.

Additional laboratory studies revealed normal liver and renal function tests, negative hepatitis panel. The biochemical parameters are enlisted in Table 1.

As the TSH levels were not suppressed, only raised total T3 level raised the suspicion for thyroid binding protein abnormality. To eliminate the possible interference generated by endogenous antibodies from multiple myeloma, serum of the patient was mixed with an equal proportion of polyethylene glycol (PEG) 6000, i.e., 200 *μ*l. Alongside, a normal control serum was also precipitated with the PEG to ensure that the T3 was not precipitated. The solution was incubated at 37°C during 10 min and then centrifuged for 10 minutes at 5000 rpm. Total T3 activity was reassessed within the supernatant, and result was 0.82 nmol/L (reference range 0.6–2.79), i.e., within the normal reference range. Furthermore, the IgG levels which were 113.45 g/L (normal range: 6.5–16 g/L) at baseline declined to 0.74 g/L (normal range: 6.5–16 g/L) posttreatment with PEG.

However, the anti-mouse antibody blocking analysis and the linearity study with dilution to confirm the interference could not be carried out because we did not have enough baseline serum of the patient to carry out the analysis. However, the patient stated that he had no previous exposure to mice.

**Figure 1:**
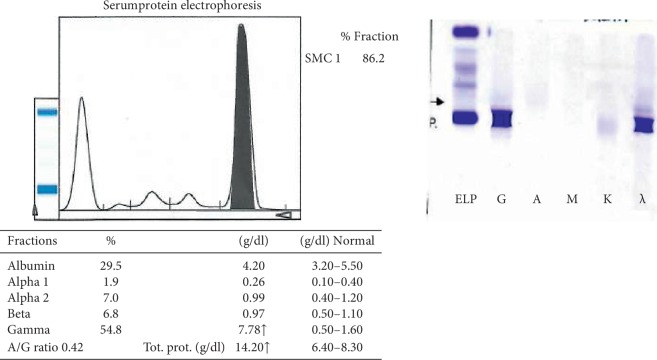
Serum protein electrophoresis showing the M-spike and serum immunofixation (IFE) results consistent with IgG lambda monoclonal gammopathy.

Serum protein electrophoresis (SPE) was undertaken which showed gamma globulin of 7.78 g/dL (0.5–1.6) with an M-spike, consistent with diagnosis of multiple myeloma and serum immunofixation (IFE) revealed IgG lambda monoclonal gammopathy as shown in (Figure 1).

Bone marrow examination showed cellular areas with diffuse infiltration with plasma cells (90–95%). Plasma cells positive for CD138 and CD56. Skeletal survey was positive for multiple lytic lesions. All findings were consistent with diagnosis of multiple myeloma.

As the patient was clinically and biochemically euthyroid, total T3 was falsely elevated which was determined to be due to the excess gamma globulins interference in serum with T3 only, and the case was further referred to hematologist for appropriate management. The patient underwent treatment for multiple myeloma as per the recommended protocol based on cyclophosphamide, bortezomib, and dexamethasone. The IgG levels which were 113.45 g/L (normal range: 6.5–16 g/L) at baseline normalized to 11.35 g/L (normal range: 6.5–16 g/L) posttreatment. Furthermore, the total T3 levels posttreatment were 1.25 nmol/L (reference range 0.6–2.79).

## 3. Discussion

Nearly, more than 99% of both total T3 and T4 are bound to either the thyroxine-binding globulin (TBG), transthyretin (TTR), or albumin. The remnant fraction is the hormonally active free hormone [3]. The routinely used total T3 and T4 immunoassays quantitate both the free and bound forms. Keeping this fact in mind, any degree of change in these binding proteins will result in change in the serum concentration of total T3 and T4, generating spurious results creating a dilemma for diagnosticians in the presence of normal TSH levels.

TBG abnormalities are usually suspected the cause when an endocrinologist encounters a patient with elevated T4 or T3 with normal TSH and the free forms of the hormone. TBG excess is primarily hereditary or it can be secondary to excess estrogens, hepatitis and certain medications 5-fluorouracil, opiates. [4, 5]. In our case, the history does not comprise any medication known to alter TBG levels alongside a negative hepatitis panel. Furthermore, hereditary excess TBG production was ruled out as the patient had previously normal thyroid function tests. Additionally, autoantibodies to T3 or T4 have also serve as thyroid hormone binding proteins and can falsely alter the levels of T3 and T4 [6]. However, as the prevalence of anti-T3 or anti-T4 antibodies among healthy individuals is very rare, it was not further evaluated.

**Figure 2:**
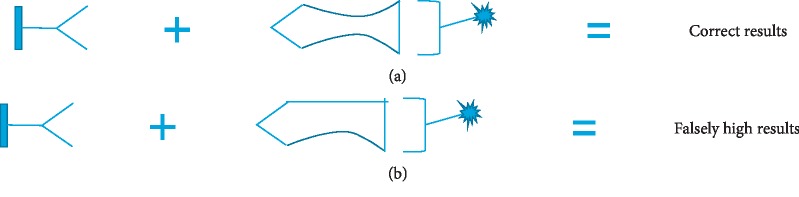
Crossreactions by interferents in two-site immunoassay. (a) Antibodies bind to specific analyte: correct result obtained; (b) cross-reactant sharing two epitopes in common with analyte: falsely high results obtained.

In the 90s, a case of factitious hyperthyroxinemia, due to a monoclonal IgA kappa multiple myeloma was reported with falsely elevated total T3 and T4 not correlating with a normal TSH and free form of both the hormones [7]. In our case, there was absence of any clinical evidence suggesting abnormal TBG, so the high total T3 is most likely justified by the monoclonal immunoglobulin identified on serum protein electrophoresis and immunofixation, binding T3 with high affinity but not T4, as T4 levels were within normal limits. Furthermore, as the Advia Centaur immunoassay is a competitive binding assay, the tracer total T3 binding to the assay antibody follows an inverse relationship with the patient's total T3 levels, i.e., it will eventually decline as the patient's serum T3 levels go up (Figure 2). In the scenario of the monoclonal antibody interfering with the tracer binding, a significantly elevated total T3 would be expected. This is based on the fact that the T3 including the tracer T3 would bind more poorly. Based on this phenomenon, an erroneously elevated T3 was evident in this case. Since the concentration on the architect was also elevated, but other immunoassays based on different working principle were not explored and the possibility of interference with the other assays cannot be ruled out.

To the best of our knowledge, till date, only one similar case with interference with only T3 has been reported by Antonopoulou et al. with euthyroid hyper-tri-iodothyronemia. In this case, the high total T3 was revealed to be due to the high monoclonal immunoglobulin level, binding T3 with high affinity but not T4, as evident by normal T4 levels revealing a diagnosis of multiple myeloma [2].

## 4. Conclusion

Our case report is only the second till date suggesting that monoclonal immunoglobulins from multiple myeloma may exhibit binding with higher affinity to T3 only leading to falsely elevated T3 levels in euthyroid patients. It is noteworthy to recognize that spurious results of total thyroid hormones in presence of normal TSH could be due to interferences and requires thorough evaluation based on appropriate clinical history and laboratory workup.

## Figures and Tables

**Table 1:** Biochemical workup.

Hemoglobin	9.1 g/dl (normal range: 12.3–16.6 g/dl)
Hematocrit	28.9% (normal range: 38.4–50.7%)
White blood cell count	7.8 × 10^9^/L (normal range: 4.8–11.3 × 10^9^/L)
Platelets	296 × 10^9^/L (normal range: 154–433 × 10^9^/L)
IgG	113.45 g/L (normal range: 6.5–16 g/L)
Serum creatinine	0.9 mg/dl (normal range: 0.9–1.3 mg/dl)
Beta-2-microglobulin	6020 ng/ml (1210–2700 ng/ml)
Serum calcium	9.9 mg/dl (8.6–10.2 mg/dl)
